# Tranq burn: Exploring the etiology of xylazine-related soft tissue injuries

**DOI:** 10.1016/j.drugpo.2025.104830

**Published:** 2025-05-08

**Authors:** Daniel Ciccarone, George Karandinos, Alex Krotulski, Jeff Ondocsin, Nicole Holm, Fernando Montero, Max Denn, Christopher Moraff, Sarah Mars

**Affiliations:** a Family and Community Medicine, University of California San Francisco, 490 Illinois Street, Box 0900, San Francisco, CA 94158, USA; b Division of General Internal Medicine, Massachusetts General Hospital, 100 Cambridge St., Suite 1600, Boston, MA 02114, USA; c Harvard Medical School, 25 Shattuck Street, Boston, MA 02115, USA; d Center for Forensic Science Research and Education, Fredric Rieders Family Foundation, 206 Welsh Road, Horsham, PA 19044, USA; e HIV Center for Clinical and Behavioral Studies and Social Intervention Group (SIG), Columbia University, 722 West 168th Street, NY, NY 10032, USA; f Narcomedia, Inc., Philadelphia, PA, USA

**Keywords:** Xylazine, Tranq, Skin and soft tissue infections, Skin and soft tissue injury, Injection, Fentanyl, Synthetic opioids, Philadelphia

## Abstract

**Introduction:**

‘Tranq dope’ is a combination of xylazine and fentanyl that is increasingly common in the US. Frequently injected, its use appears related to severe skin and soft tissue wounds (SSTW) through an unknown mechanism. Previous research suggests that the high acidity of certain heroin source-forms contributes to vein damage and SSTW, however, the possibility of a role between the acidity of tranq dope and SSTW is understudied.

**Methods:**

A convenience sample of persons who use drugs participated in semi-structured interviews (Philadelphia, Oct. 2023, *n* = 30). Observations of wounds/injection locations were made. We analyzed narrative data for perceptions of wound causation. Our partner lab analyzed the pH of 10 independently obtained samples, including tranq dope (*n* = 4), street opioids without xylazine (*n* = 2), xylazine alone (*n* = 2), and street stimulants (*n* = 2).

**Results:**

Observed SSTW were extraordinarily severe. Several themes emerged related to wound etiology: 1) tranq dope injection caused burning sensations; 2) vein loss occurred rapidly following uptake of tranq dope; 3) vein loss resulted in increased injection attempts, the use of large central veins (e.g., jugular and femoral), as well as more frequent ‘skin-popping’; and 4) wounds (called ‘tranq burn’) rapidly followed vein loss. The average pH of the samples was 4, with samples containing fentanyl ranging from pH 2.1–5.9; samples containing xylazine ranging from pH 3.6–5.9; and the cocaine sample with a pH of 3.

**Discussion:**

While this study cannot confirm a causal role, our findings of reported burning sensations and moderate to high acidity of lab-tested drugs are coherent with reported rapid vein loss following initiation of tranq injection. This, in turn, lends early support to a synergistic hypothesis of tranq-related SSTW etiology: vein loss and subcutaneous injections stem from the injection of acidic drugs followed by poor tissue perfusion from vasoconstriction due to xylazine. Possible harm reduction interventions include dilution and buffering. Stigma reduction and enhanced wound care are required in harm reduction and clinical settings.

## Introduction

The 20-year US opioid overdose epidemic, marked by waves of mortality (Ciccarone, 2019; Ciccarone, 2021), is striking in that the most recent wave involves intentional or unintentional co-use of opioids with another category of substance (Ciccarone et al., 2017; Ondocsin, Holm, Mars, & Ciccarone, 2023; Mars et al., 2024). The widespread introduction of xylazine into the US street fentanyl supply – the combination commonly called ‘tranq dope’ – represents the most recent large-scale transformation in the unregulated drug supply. Xylazine is an alpha-2 adrenergic receptor agonist that is used in veterinary medicine as a sedative but is not approved by the US FDA for human use.

Xylazine first emerged in the illicit drug supply in Puerto Rico in the early 2000s, where it was locally known as *anestesia de caballo* (horse anesthetic) and sold alongside street heroin, and later pre-mixed into the drug (Torruella, 2011). These early reports found that it was commonly injected in a speedball (cocaine & heroin) and that persons who use drugs (PWUD) quickly identified it as a veterinary product, were concerned about becoming addicted to a separate non-opioid substance, reported sleeping more, and noticed an uptick in ulcerative skin wounds (Reyes et al., 2012).

Xylazine has been a periodic adulterant in the US drug supply for over a decade, but its presence has accelerated significantly since the late 2010s (Friedman et al., 2022). First detected in Philadelphia overdose deaths in April 2006 (Wong et al., 2008), xylazine was detected relatively rarely in fatal overdoses between 2010 and 2015 (<2 % of cases) before being identified in >30 % of decedents in 2019 (Johnson et al., 2021). Xylazine’s contributions to the ongoing US overdose epidemic (Ciccarone, 2019; Ciccarone, 2021) remain unclear, although a multi-center study of specific health outcomes (cardiac arrest and coma) in emergency department patients presenting for illicit opioid overdose found lower odds of these outcomes among those testing positive for xylazine (Love et al., 2023).

National Forensic Laboratory Information System (NFLIS) data from 2019–2022 found that the percentage of drug reports positive for xylazine increased in all but three states, doubling in 30 states across all US regions (Cano et al., 2024). Despite this expansion across the US, xylazine remains particularly problematic in the US Mid-Atlantic and Northeast (Gupta, Holtgrave, & Ashburn, 2023; Spencer et al., 2023). By 2021, xylazine was present in over 90 % of street opioid samples in Philadelphia (Substance Use Philly, 2024), while data from the first half of 2023 found xylazine present in 99 % of purported fentanyl samples (Center for Forensic, 2023). During this same period, the proportion of xylazine present in street opioids in Philadelphia has also increased. Quantitative analysis of street opioid samples over 2022–2023 found that the average amount of fentanyl in each sample has remained relatively consistent while xylazine content has risen, from 34.3 % purity/weight in Q3 2022 to 46.1 % in Q2 2023 (Center for Forensic, 2023).

The issue of skin and soft tissue wounds (SSTW) has become one of the primary consequences of xylazine-fentanyl use with their prevalence, persistence, and severity, particularly of clinical concern (Malayala, Papudesi, Bobb, & Wimbush, 2022). Tranq dope-associated wounds frequently begin as small blisters or bumps and then progress to necrotic skin ulcerations that may affect sizeable areas of skin, fat, and muscle, and in their most severe state, reach full-thickness necrotic damage down to tendons and bone, disfiguring and disabling limbs and sometimes resulting in amputation (Carroll, 2024; McFadden et al., 2023). Studies drawing on the experiences of harm reduction workers and nurses managing tranq-related consequences have reported that wounds develop rapidly, emerge both at and distal from injection sites (Carroll, 2024; McFadden et al., 2023; Zagorski et al., 2023), occur among individuals using non-injection routes of administration (McFadden et al., 2023), and can be challenging to treat, particularly among unhoused populations without access to running water or stability for changing dressings (Carroll, 2024; McFadden et al., 2023; Zagorski et al., 2023). These experts were, however, confident that xylazine wounds could be treated successfully without the need for widespread amputations (Carroll, 2024; McFadden et al., 2023; Zagorski et al., 2023).

The mechanism by which xylazine contributes to these wounds and vein loss has not yet been elucidated. Xylazine, an alpha-2-agonist, is structurally similar to clonidine, causing sedation and cardiovascular side effects, including bradycardia, hypotension and cardiac arrest (Ruiz-Colón et al., 2014), as well as peripheral vasoconstriction. Tissue necrosis is thought to be multifactorial, including direct damage from injections and indirectly through poor tissue perfusion. Other posited contributions include xylazine-induced vasoconstriction and impairments in wound healing secondary to an unknown mechanism, possibly related to nitric oxide synthase activity (Reagan-Udall Foundation for the FDA, 2024). A recent mouse-model study found that xylazine is a full kappa opioid receptor agonist and exhibits morphological similarities in wound development to those seen in historical outbreaks of other kappa opioid agonists (Bedard et al., 2024).

Ciccarone and Harris (2015) posit that venous sclerosis is a proximal risk factor for skin and soft tissue injuries and infections. They proposed that differential causticity/acidity of heroin source-forms is a factor in the development of venous sclerosis among some populations of people who inject heroin. In their hypothesis, injection of caustic drugs led to accelerated vein loss and perivenous and muscle injections, setting up the nidus for infections. In their pilot study of heroin acidity carried out in Philadelphia and London, the mean pH of Philadelphia street heroin was 4.7, with speedball preparations registering a mean pH of 4.2 (Ciccarone & Harris, 2015), acidities in between orange juice and coffee.^1^ In contrast, London samples where heroin required the addition of an acid (ascorbic or citric acid) to go into solution had a 1–2 log order lower pH, ranging from 2.1 to 3.5, with a mean pH of citric acid-prepared heroin of 2.6, similar to that of vinegar (Ciccarone & Harris, 2015). London PWUD suffered from severe venous damage, with resultant leg edema and a variety of moderate to severe skin and soft tissue injuries and infections. PWUD in Philadelphia, by contrast, had few venous and skin issues unless they regularly injected speedballs.

The possibility that tranq dope use is also leading to accelerated venous sclerosis—scarring of veins and loss of functionality—and SSTW requires further exploration. These injuries are most frequently the result of accidental missed injections (Ciccarone et al., 2000), as well as intentional “skin-popping” (injecting subcutaneously) or “muscling” (injecting deep into the muscle) injections after losing easy peripheral vein access. Following Ciccarone and Harris, we offer an additional mechanism of injury that may contribute to both venous sclerosis and tranq dope-related wound development: the acidity of injected street solutions.

This paper results from a multimethod study combining ethnographic interviews and observation of street substance use with laboratory testing by the Center for Forensic Science Research and Education (CFSRE) of the acidity of drug samples collected in the Kensington neighborhood of Philadelphia. Drug samples were sourced from CFSRE’s library of submitted samples for drug checking in Philadelphia. We explored the hypothesis that the injection of potentially caustic substances (fentanyl, xylazine, cocaine, and methamphetamine) is contributing to the development of the now widespread tranq dope-related wounds among PWUD in Philadelphia.

## Methods

This study used rapid ethnographic assessment to examine xylazine polysubstance use in Philadelphia, Pennsylvania (Fessel et al., 2019). A semi-structured interview guide was drafted before the trip, and the research team (DC, GK, FM, NH, and JO) gathered in Philadelphia for one week in October 2023. One author currently lives in Philadelphia, while three others had previously lived in the city for multiple years while conducting research on drug markets and patterns of use in Kensington (Bourgois, Hart, Montero, & Karandinos, 2018; Friedman et al., 2019; Karandinos, Hart, Montero Castrillo, & Bourgois, 2014). The PI has more than a decade of experience conducting research in Philadelphia (S. Mars, Bourgois, Karandinos, Montero, & Ciccarone, 2014; S.G. Mars et al., 2015; S.G. Mars, Bourgois, Karandinos, Montero, & Ciccarone, 2016). The first day consisted of pilot research in the Kensington neighborhood of Philadelphia to observe the street-based risk environment, recruit potential participants for future research days, and pilot the initial interview guide. The interview guide was revised in an iterative process based on observations and interviews from the first day by all team members and was used for the subsequent fieldwork days. Changes to the interview guide consisted of reducing its overall length, combining questions that yielded overlapping or contradictory answers, and identifying “key” questions that were particularly essential to the project. Interviews lasted 30–90 min, and topics of discussion included substance use trajectories and preferences, the emergence of xylazine in the drug supply and subsequent changes in modes of use or use frequency, history of and experiences with xylazine-related wounds, and trends in the local drug markets. Interviews were conducted in pairs as much as possible to include the perspectives of multiple researchers and ensure the safety of the research team. However, due to time constraints and team availability, some interviews were conducted by only one interviewer. Some participants consented to photography of drug preparation and consumption or of wounds, with a few additionally participating in video-recorded consumption sequences. Participants were compensated $25 for interviews and $25 for a video-recorded sequence. Daily collaborative field notes supplemented interview and photographic data.

Recruitment was predominantly street-based in Kensington, with some snowball sampling from participant networks and personal contacts of one of the authors (CM) from other parts of Philadelphia. Some participants were referred to the research team by community partners who provided street-based outreach in Kensington. Interviews were conducted in locations adjacent to recruitment sites upon consultation with participants, including public parks, the rental cars of the research team, and participants’ homes or shelters. These interviews took place at some distance from other individuals to ensure privacy. Participants were aged 18 years or older and self-identified as a person who used drugs. Members of the research team read aloud the informed consent to each potential participant, and consent was obtained verbally to protect participant identities. Participants who were intoxicated or otherwise incapable of consenting were excluded from participation. The University of California, San Francisco Human Subjects Protection Program approved the research methodology, and a National Institutes of Health Federal Certificate of Confidentiality protects the data. The demographics of the sample can be seen in Table 1.

Interviews were audio recorded and professionally transcribed in their entirety before being checked for accuracy by the authors. The team drafted analytic memos based initially on the interview guide for each transcript and met weekly to discuss emergent themes. A thematic memo based on these findings was subsequently drafted to analyze participants’ experiences with and beliefs about xylazine wounds, which served as the basis for this manuscript. All names included in this manuscript are pseudonyms.

### Lab pH testing

Drug materials (e.g., pills and powders) were received from public health collaborators as part of larger drug checking and surveillance initiatives. Samples were obtained from the repository of CFSRE’s drug checking database. A convenience sample was obtained for this study. Firstly, we selected samples obtained from the same neighborhood (Kensington) and time period (past 3 months) of the ethnographic study and that were of sufficient quantity (> 20 mg). Four categories of drug combinations were preselected (heroin/fentanyl only, xylazine plus fentanyl, reference samples of xylazine, and stimulants. Within each category, 1– 4 samples were randomly selected. Routine qualitative analysis was performed by gas chromatography-mass spectrometry (GC–MS) and liquid chromatography quadrupole time-of-flight mass spectrometry (LC-QTOF-MS). Resulting data files were processed against an in-house library database of >1100 targets. Samples with enough mass (i.e., 5–10 mg) were aliquoted for quantitative analysis performed by gas chromatography-mass spectrometry (GC–MS) using a directed scope that included fentanyl, xylazine, and other substances, and results were reported as a weight-by-weight percent. To determine the pH of samples, full glassine bags containing “dope” powder were transferred to a test tube, and 150–600 uL of water (pH 7.1), depending on the weight of the sample, was added before taking the pH measurement using a lab-grade pH meter.

## Results

Warning: This paper includes graphic images of xylazine-related wounds.

### Emergent hazard

*We’re using animal tranquilizer, for God’s sake. Like, this shit is putting holes in our body that you could stick your hand through. I’ve seen people who walk around with exposed bone*. (Amber, 37yo, injecting tranq)

Xylazine is dominant in Philadelphia’s street-opioid scene today, following a similar trajectory to the introduction of fentanyl into the US heroin supply: first introduced as a limited adulterant before wholly overtaking the market and supplanting fentanyl as the primary component in the city’s ‘dope’. The combination of xylazine with fentanyl is termed ‘tranq dope’ or sometimes simply ‘tranq’, although at times ‘tranq’ is used to refer solely to xylazine:

Interviewer: What really is meant by tranq dope?

Michael: You do it and you’re knocked out.

Interviewer: What is meant by it chemically? What is the actual drug?

Michael: …Without the xylazine, there’s no tranq; there’s no tranq in the dope.”

(Michael, 47yo, injecting and smoking tranq)

Participants were nearly unanimous in their disdain for xylazine. Chris, 23yo and injecting tranq dope, described the addition of xylazine as representing a decline in drug quality with severe social and health consequences:

The quality of drugs down here has been so far downhill. It’s, a lot of the time, it isn’t real. It’s all like synthetic shit. […] I think we really just need to get this xylazine off the street. It’s fucking destroying people’s bodies. You can’t even take your kids on public buses because all you smell is the rotten flesh from the xylazine. I’m 23 years old, and I’m having liver and kidney failure. I got holes in my legs, my arms. I can barely walk and get up some mornings.

(Chris)

Interviewees indicated they were dependent on both fentanyl and xylazine, and using street opioids outside of Kensington would not help them manage withdrawal symptoms because it lacked xylazine: *It’s nothing that I get excited over. It’s just something that needs to be there now. […] …I’ve tried to do just… plain fentanyl. And I would get well from the fentanyl but not well from the xylazine*. (Daniel, 32yo, injecting and smoking tranq)

The introduction of xylazine was recognized as marking a new era for the widespread development of extensive vein damage and skin and tissue wounds notable enough to merit the coining of a new term, ‘tranq burn’. While it was well established among participants that the injection use of cocaine was damaging to veins—“*Anybody know that it’s the powder* [cocaine] *that messes up* – *that’s what mess your veins up, too. […] the powder has been known forever to do that”* (David, 35yo, injecting tranq)—and prior research in Philadelphia found that injecting cocaine alongside opioids resulted in more significant vein loss than heroin injection alone (Ciccarone & Harris, 2015; Mars et al., 2016; Mars et al., 2015), long-time users of opioids were surprised by the effects of tranq dope on their vein health:

Interviewer: Have people always had this much trouble injecting, or is this newer?

Michelle: No, no. I don’t think so. I don’t think that the drugs have been so noxious to the veins, either. Powder [cocaine], yes. We all know powder kind of destroys your veins, but we really didn’t know – or at least I wasn’t aware that tranq would be just as bad, if not worse, than powder shots.

(Michelle, 49yo, injecting tranq)

Many participants indicated that their vein health had rapidly deteriorated since the emergence of tranq dope:

Interviewer: So, you’ve been using tranq for about two years, then?

Heather: Yeah, about two years. But yeah, when I came here, it got a lot worse, though. Like, I just wanted to keep shooting it and shooting… And tranq fucks you up. …you can’t shoot up anymore. […] Your veins get fucked up… It’s hard. [Participant shows interviewer where she injects in her arms] Feel that [scarred vein]…. That’s what it does.

(Heather, 33yo, injecting, skin-popping, and smoking tranq)

They reported needing more time to perform injections, requiring more ‘pokes’ to register in veins and veins hardening and becoming inaccessible over time. While spending time with participants, we witnessed many instances where people performed multiple pokes before registering in veins and often seemed to check several times while injecting that they remained in the vein, sometimes resulting in another flurry of poking.

Venous access issues and transitions from peripheral to central veins (e.g., jugular, femoral) are somewhat common among people injecting drugs over the long term. Still, in our observations, we witnessed a striking number of people injecting, or being injected, into their jugular and brachial veins. Participants described wearing out peripheral veins quickly and beginning to inject into larger veins, with potentially dangerous consequences: *“I used to inject in my neck, but I’m scared to go back in my neck. Because I’m scared to get hit in a hot vein* [accidentally injecting in an artery]*.”* (John, 25yo, injecting and snorting tranq). The introduction of tranq dope has accelerated this process, and participants resorted to central veins or alternate modes of use after only weeks or months of using tranq:

Interviewer: Tell me about those [point to large arm wounds]. How did you get them? How long have they been there?

Justin: Oh, man. I’ve had them for about three years now. …I used all my veins up from shooting the xylazine. It tears your veins apart. So, I’ve gone through every single vein you can dream of in your body from feet, from head to toe, neck, everything.

Interviewer: Now, some people have gone into their necks and arms—underarms. How come you didn’t do that?

Justin: I did. The veins in my neck were gone. I couldn’t even get a vein in my neck.

Interviewer: And how long were you injecting tranq before your veins all disappeared?

Justin: Probably two or three months.

(Justin, 27yo, injecting, muscling, and skin-popping tranq)

Transitioning completely or situationally to alternate injection modes, e.g., “skin-popping” and “muscling,” was sped up in some instances by the development of tranq wounds at preferred injection sites. Justin, however, indicated that the switch to skin-popping had not resulted in substantive improvement in his wounds but had accelerated their growth and introduced further skin and tissue damage that he continues to manage:

Justin: …So, I started skin-popping [tranq]. And when you skin pop, you know, you’re not hitting a vein. You’re just putting it right under the skin. So, once you do that, it just eats from underneath the skin to the top of your skin. And it just falls off.

Interviewer: How long were you skin-popping before the wounds got this bad?

Justin: Two weeks.

Other participants reported that their skin and muscle tissue had hardened and darkened since initiating tranq dope use, further limiting injection options: *My legs were really bad. I stopped in my legs, started going in my arms. Because my legs were like rock hard, they were starting to get darker. So I didn’t know if they were like dying inside*. (Ashley, 37yo, injecting, skin-popping, and muscling tranq)

Some initiated non-injection modes of use, e.g., snorting or smoking, due to vein loss as well as other reasons, including lowered tolerance after periods of sobriety or rehab, fears about using syringes, and concerns about overdose:

Interviewer: Do you ever smoke anymore?

Taylor: Oh, yeah, yeah. Actually, I keep a bubble on me at least on me at all times because sometimes [I] can’t hit.

Interviewer: So you can’t hit, and then you just have to smoke because you don’t have an option?

Taylor: Yeah.

(Taylor, 26yo, injecting and smoking tranq)

Interviewer: So have you always smoked the dope, or did you—

Reggie: No. I used to snort it before. I never shot it because I was too scared. I was scared that, you know, that’s why a lot of people die, like overdoses. The majority of overdoses come from people who inject it. It’s easier to overdose that way than smoking it.

(Reggie, 42yo, smoking tranq)

Additionally, respondents believed that, in notable contrast with the opioids they had used before in Philadelphia, tranq dope could be smoked, albeit with some risk of inefficiency or drug loss: *“…I think the damage the xylazine was doing to people’s bodies kind of led them to smoking it. And, also, the dope before, you couldn’t smoke a lot of it. Even now, you can’t smoke [all] of the dope. So, smoking it’s a risk [for missing a dose].”* (Chris)

While recent studies have shown encouraging results in overdose reduction among those who smoke fentanyl relative to injecting it (Karandinos et al., 2024; Megerian et al., 2024), it is less clear that smoking tranq is a route to avoid wound development. Interview participants who smoked did indicate they were less troubled by wounds—including the extensive tranq burn experienced by people who inject—but were not wholly immune. Since first snorting tranq, people reported wounds developing as well as issues with nasal tissue, with participants exhaling bloody or hardened material from their noses.

### Wound etiology

Potentially salient to tranq dope wound development were reports of ‘burning’ sensations when injected. Participants were asked to approximate how much individual drugs burned when injected or when shots were ‘missed’ [accidentally injecting outside the vein into tissue] as a possible proxy measure of drug causticness and resultant vein and tissue damage. Michael considered that tranq burned when missed but less than cocaine, while Robert, 46yo and injecting and skin-popping tranq, felt a burn even when hitting the vein:

Interviewer: How does tranq feel when you miss your vein?

Michael: It burns. It burns. Burns, burns, burns.

Interviewer: Is there any other chemical that it reminds you of in terms of the level of burning? Like if you missed a shot with coke, would it burn as much?

Michael: It burns a little less than coke.

Robert: …Even if I hit, the vein burns. Like it burns going in.

Interviewer: Does it burn afterwards or for a while, or is it just when you’re injecting?

Respondent: Just when you’re hitting, it’ll burn.

Although possibly less painful than with cocaine, ‘missing shots’ was considered particularly significant in tranq wound development and a notable distinction from the pre-tranq era. Michelle indicated that missing with tranq dope was much more likely to result in the development of an abscess or tranq wound than before:

Interviewer: If you miss with powder vs. tranq, how does that compare?

Michelle: It’s almost the same, except for when you miss with tranq the chances of it becoming an abscess are like 100 times more likely…. It’s not just an abscess. It’s just like it burns.

This terminology was reproduced in participants’ descriptions of xylazine wounds as ‘tranq burn’, differentiating the large wounds characteristic of xylazine use from abscesses and other skin and soft tissue infections participants had previously experienced. However, other participants reported that tranq dope would only burn when missed, and a minority thought that it never burned, whether intravenous injections were administered incorrectly or not. Burning sensations were not limited to intravenous injection, with reports of tranq burning when muscled: *“…So, since I’m doing muscle shots, this is the only hole I had gotten. It burned crazy. I thought it was a chemical burn.”* (Ashley) Combining cocaine with tranq was believed to result in an especially strong burning sensation: *More intensified. Like, if you’re putting powder in with the dope, the burn is going to be a lot more fuckin’ vicious, and it’s gonna swell up instantly*. (William, 37yo, injecting, skin-popping, and muscling tranq)

Even when tranq injections were not missed, participants noticed flesh and vein damage that preceded wound development:

Interviewer: So, all those knots are new in the last two months?

Amy: Yeah. And they’re all from where I shot tranq. And some of them weren’t even where I missed. Like this? I didn’t even miss. Like that just put a knot there after I shot it. I don’t know why. […] Like almost like it clogs my veins up and knots it up or something.

(Amy, 36yo, injecting and smoking tranq)

While participants reported that wounds were likely to develop at injection sites, this was not universal, and participants injecting in central veins were not experiencing wounds at these locations: *“[…] I inject in my neck, or like my clavicle or my groin area and I never have [wounds] there.”* (Jon, 36yo, injecting tranq)

### Wound progression

There was a standard progression of tranq dope wound development that participants understood well. They indicated that tranq wounds often started as a pimple, bubble, or hard knot before becoming the large wounds characteristic of tranq with black patches of necrotic tissue dotting the surface of the wound, areas of scabbing, and underneath the scabbing what participants described as ‘holes’ of various depth. Missed shots and burning sensations often presaged later wound development:

Anthony: It started with like a pimple. […] You would go to fucking hit in a vein and like you’d be hitting and … maybe like you finish it, and then, you realize like, oh, you went and missed like the last part. Because there’s a delayed reaction to it. Like five seconds will go by after you’re done. And then, wow, fuck, it starts like burning. And then, the next morning, you wake up and you’ll see it makes your skin—it looks like a dark bruise. It’s like a black patch. And it’s like, all right now. Fucking that’s that flesh-eating shit.

Interviewer: So it’s from a missed shot?

Anthony: Yeah. My stuff is mostly from missed shots.

(Anthony, 37yo, injecting tranq)

**Fig. 1.**
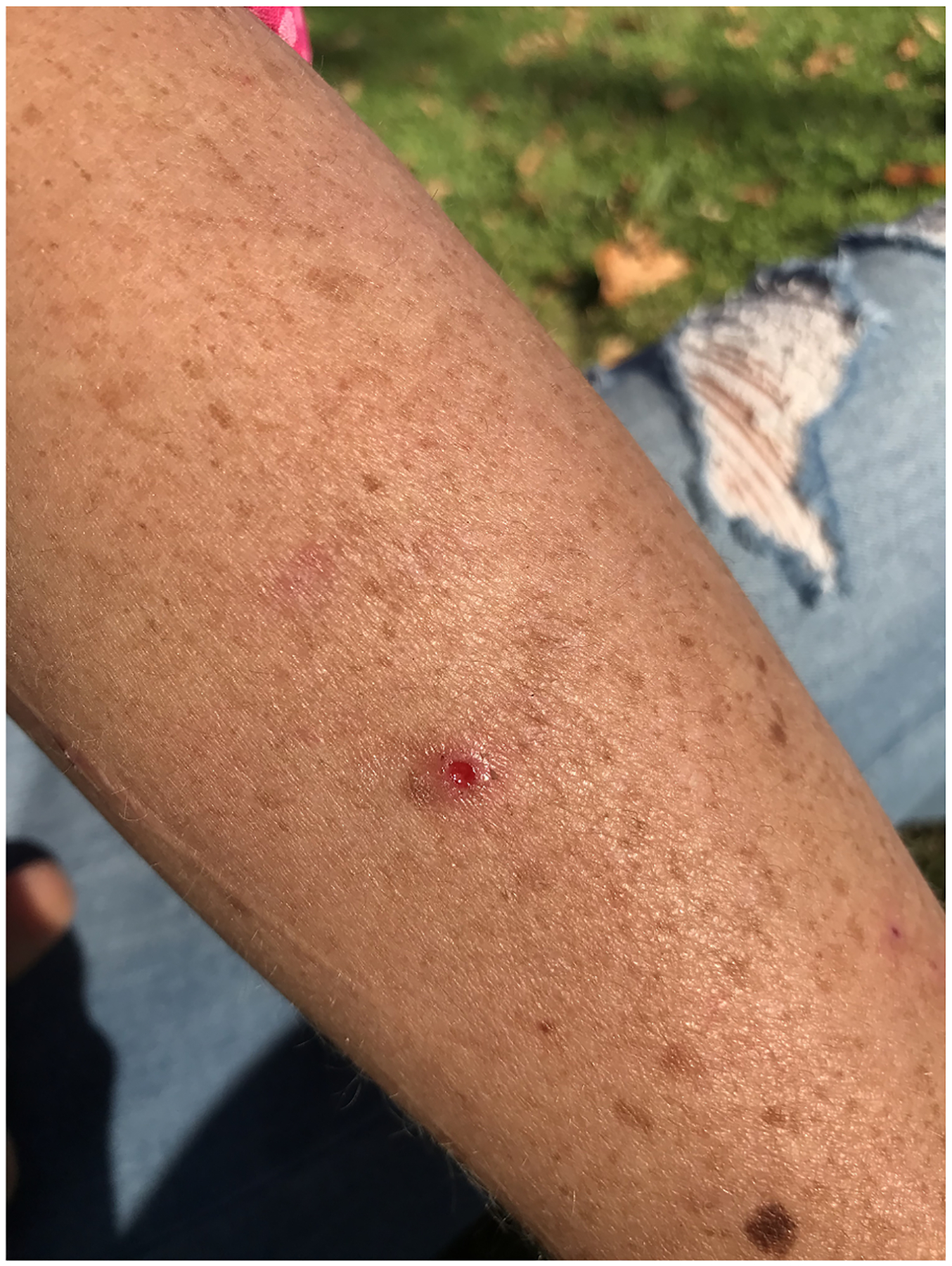
Image pimple.

Fig. 1. Even among those who tried to maintain good injection practices—rotating injection spots, keeping skin and equipment clean, and using new equipment for each injection—negotiating vein health while using tranq was a challenge. Participants maintained that, in addition to accelerated vein loss, tranq wounds developed quickly after injection and worsened rapidly, especially when they were unable to seek treatment quickly. Wounds failed to heal completely, lingering over time:

Interviewer: So, about a month after starting tranq, you started getting these –

Amy: Well, maybe like two weeks after I started tranq. And then I started to get it. And then it got bigger and bigger. And then it kind of stopped at where it’s at now, thank God. But it’s been a month now of this. Like not closing up. Like it’s not coming together at all. And what it did was it scabbed over. But then the scab fell off, and it’s still just an open – it looks exactly the same way it did before it ever scabbed over. Like it’s not physically closing.

For some, wounds had recurred in the same locations for years, improving over time or even fully healing before reemerging in the same location:

Interviewer: This is really the first one?

David: Yeah. And it been here for a long time. It’s been about a long time.

Interviewer: It was like weeks, months?

David: Four years – four years.

Interviewer: This wound has been there for four years?

David: It heal, it’ll come back, heal, come back, yup.

Interviewer: Have you got any medical care for it?

David: Originally, yeah, but not lately. Get antibiotics once in a while sometime, when I need it. Then it just never closed. It gets smaller but – yeah – opened back up.

One participant—who had many minor wounds on her lower legs—and others reported wounds emerging anywhere there was skin damage, including cuts, bruises, and injuries sustained from falls due to tranq dope’s strong sedation:

Interviewer: You have wounds on your legs? Where else?

Amber: On my arms, on my neck, everywhere. And my wounds didn’t come from needles. […] They came from me scratching myself. […] The biggest wound that I have on this leg came from me falling off of a milk crate. […] A lot of them are caused by something else.

**Fig. 2.**
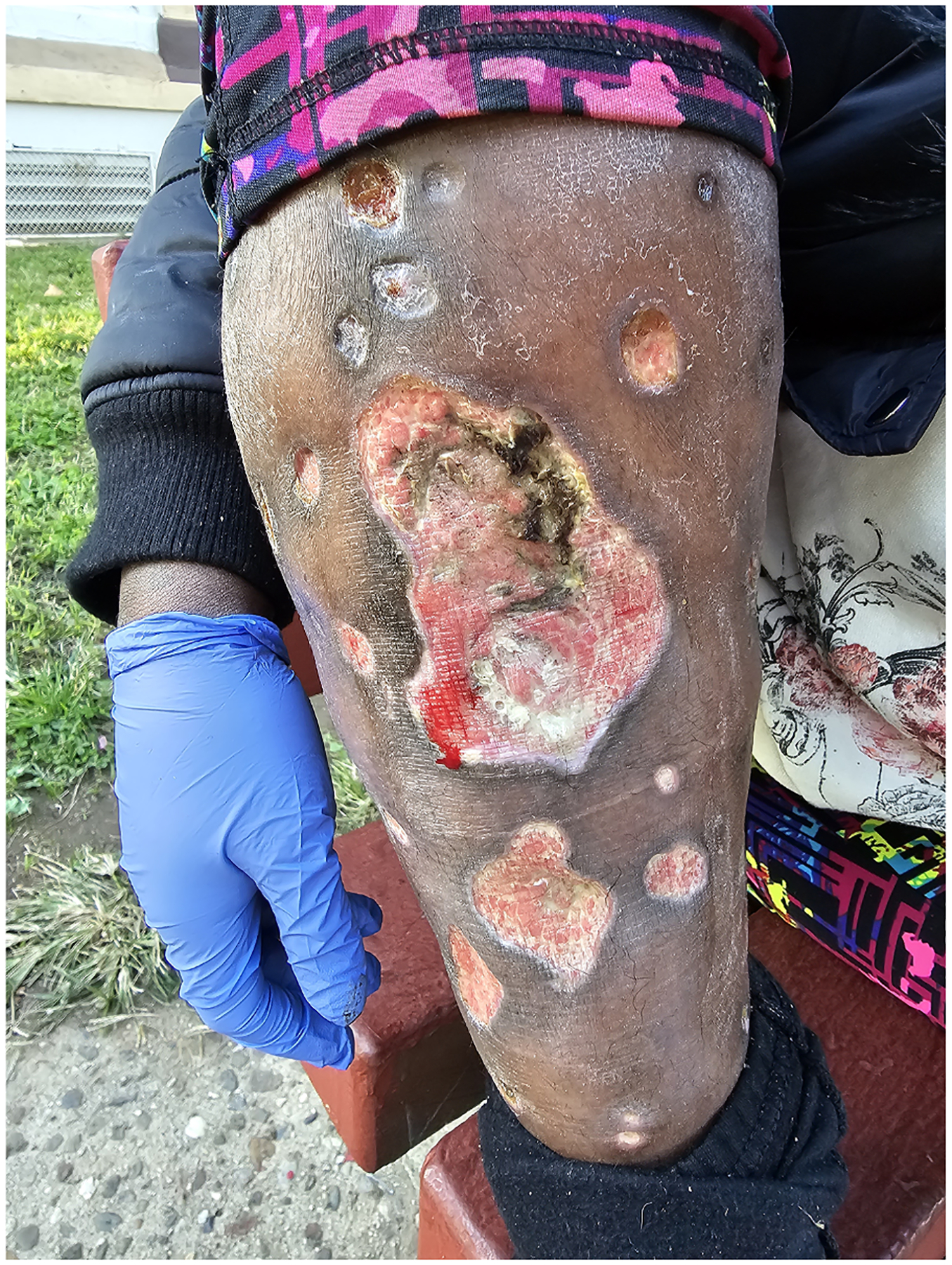
Image leg wounds.

Fig. 2 Although some indicated that tranq wounds developed away from injection sites, most participants said they were developing wounds at their primary injection location (arms or legs). Several of these individuals continued to inject directly into wounds and believed that by doing so, they were continuing to access their veins rather than skin-popping or muscling. One provided a detailed description of his process:

Interviewer: What part of the wound are you shooting into? Is it like the soft spot? […] It doesn’t make a difference if you shoot into, like, the necrotic part—that black part?

Anthony: Oh, well, no, I don’t shoot into the necrotic part and all that. […] First off, it’ll destroy the needle tip. You know? And like there’s really not much you can fucking see under there. Anything that’s not necrotic. I look for something—some of the places that are just scabbed up from having trouble hitting somewhere. I’ll just fucking flip the scab and like peel it off. And then, you’ll see like, you know, then it’s like that pus-y but fresh pink skin underneath. And, if you happen to pull off the scab where there’s a good vein underneath it, you see like flat skin with the fucking—and I like see that and I’m like, “Ah, yes.” And then, I go.

**Fig. 3.**
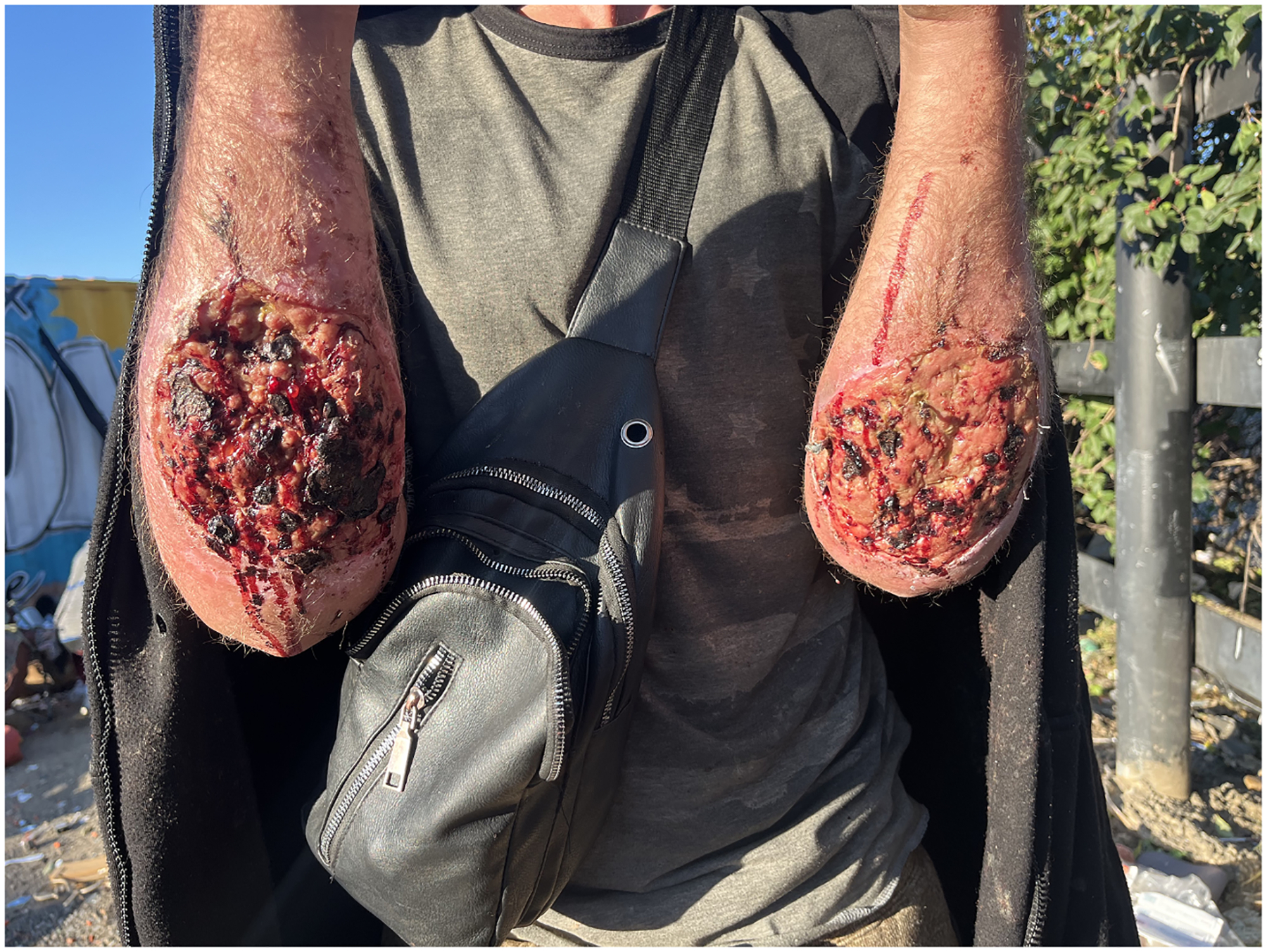
Image arm wounds.

Fig. 3 Continued manipulation of scabs and wounds likely provides new opportunities to introduce bacteria to the wound or further traumatize the skin and underlying tissue with continued poking. Among those injecting into wounds, this practice is likely a significant causal factor for wounds progressing over time. Still, participants provided several reasons for continuing to inject in wounds, including ease of access and preventing the spread of wounds to other locations on the body:

Interviewer: …If people had open veins, why would they choose to shoot in the wound as opposed to that?

Crystal: Easy target. […] It’s easier to get blood in the wound and to think that you’re in your vein than to go somewhere else and start over pricking yourself. When you already have the wound open, instead of going somewhere else and taking the chance of having another wound start, it’s just, I guess, a comfort thing….

(Crystal, 24yo, formerly injecting, currently ingesting tranq)

Registering what looked like blood reinforced participants’ decisions to continue to inject into wounds, e.g., the presence of red fluid in the syringe often gave the illusion of a venous register. While video recording an injection sequence into a tranq burn on a participant’s right lower leg, he was repeatedly unsure whether he was in a vein and, after backloading his blood- and tranq-filled solution into a second syringe, out of frustration, he eventually skin-popped his shot into the wound.

Whether injecting into wounds or in other locations, participants believed there were inherent advantages to continuing to inject intravenously, including achieving a desired effect from tranq. Muscling and skin-popping were less appealing means of using tranq because they provided little or no rush:

Interviewer: Are you muscling it, skin popping it, or going in a vein?

John: Obviously, going in a vein. […] I wouldn’t be having a full-on rush if I wasn’t—I mean, maybe I’m going into the tissue or muscle. But I won’t get a rush if I was just going into that. I’m obviously hitting some type of vein.

Others believed skin popping and muscling prevented the body from healing from the effects of tranq and were themselves to blame for tranq wound development:

Interviewer: Do you ever muscle or skin-pop?

Joseph: …Back when it was real heroin, yeah. […] Now it’s too dangerous. You can’t do that shit. It’s fucking—it deteriorates. It doesn’t allow your body to heal. You know, the tranq, it pulls all the oxygen from the blood and everything… That’s why everybody’s starting to get all them holes and, you know, tranq wounds and stuff.

(Joseph, 32yo, injecting, smoking, and snorting tranq)

Others expressed little hope for preventing tranq wound development, which they saw as an inevitable result of persistent use:

Interviewer: Why do you think some people aren’t getting wounds?

Michelle: I don’t think that they’ve done it long enough or for a long enough time. It’s going to happen eventually. You’re going to run your body down. Your body’s eventually going to go say, “Fuck you.” If it’s not done in moderation, it’s going to show its effects like anything else. Like if you drink excessively, your liver is going to show signs of it. It’s going to happen. If it hasn’t happened, it’s just a matter of time if you continue to do it…

(Michelle)

### Adding stimulants

The addition of cocaine to tranq dope may be of significance in the development of wounds due to its potential to increase the acidity of injected mixtures and its vasoconstrictive effects. The uptake of stimulant-type drugs among primary opioid users has been documented in many US locations in recent years (Ciccarone, 2021; Ondocsin, Holm, Mars, & Ciccarone, 2023; Ellis et al., 2018). Ethnographic comparison suggests that the use of cocaine speedballs, as well as methamphetamine ones, has become more prevalent in Philadelphia in recent years: *“And then doing it in speedballs with the powder, which is like right now, that’s the thing… That’s what they do around here. […] Like buying powder with your dope is like a thing now. People want a speedball. Even meth and dope.”*

(Heather)

Dealers were selling tranq as well as cocaine, while methamphetamine was estimated to be sold by a third of the dope corners. Participants noted some of the same reasons for adding stimulants to their tranq use that have been encountered elsewhere, including protection from victimization:

[…] It’s almost kind of rare to find somebody who’s just doing fentanyl now. Pretty much everybody is doing crack or coke or meth for multiple reasons. One is to stay awake so you don’t get robbed during the day, and so you can do what you got to do. Because that tranq – the xylazine just wipes you out. It just wipes you out. And then, there’s not much of a rush to it.

(Chris)

Most participants reported that tranq dope did not have any rush, perhaps because they had already become tolerant to the effects of the fentanyl component, and the addition of cocaine to the injection was newly essential for providing a desired effect:

Everyone does speedballs out here now, for the most part. Because the fetty and the shit, like I said, […] you don’t really get like the greatest rush. So, with the speedball, because the cocaine introduces the fucking rush that we’re feeling, and then, the dope and the xylazine kick in after a couple minutes—hopefully, kick in after a couple minutes. And then, it brings you down.

(Anthony)

Like other US locations seeing increased opioid-stimulant co-use, the dynamic between tranq dope and stimulant co-use is contributing to a feedback loop where each drug is used to counter adverse or unwanted effects from the other. But unlike other locations, wound development precipitated by tranq may be exacerbated by both fentanyl and the addition of stimulant-type drugs, particularly cocaine, to injection repertoires.

Over time, diminishing euphoric effects from opioids lead many to add stimulants to potentiate the duration and effects of their opioids. Participants also noted some strategic advantages of adding stimulants to their drug use repertoire to continue their use of tranq dope. These included techniques to promote venous access, e.g., by smoking crack cocaine:

…The hardest time for me to hit is first thing in the morning… Nope. Not going to be able to hit. Don’t even try it. Like everything’s dead. My heart rate is just—everything’s, you know, asleep. […] The only way I’ve been able to hit recently is I got to buy a fucking [crack] rock, take a hit just to get my blood pressure up and my heart rate up and everything so my veins will start showing. It’s terrible.

(Joseph)

### Lab pH results

The findings for the laboratory pH testing of street samples are listed in Table 2. Acidity varied by weight, volume, and composition of samples. Samples containing fentanyl with or without xylazine ranged from pH 2.1 (highly acidic, e.g., like vinegar) to 5.9 (mildly acidic, e.g., like coffee). Samples containing xylazine, in addition to fentanyl, were also acidic, ranging in pH from 3.6 (e.g., like orange juice) to 5.9, but appearing perhaps less acidic than fentanyl samples without xylazine. Xylazine acidity may vary by source, speculatively due to differing salt-forms (e.g., HCL). Sample 5, with a pH of 3.9, approximated the average fentanyl and xylazine purities found in street samples at the time of the ethnographic research; its weight also approximated the average per-use weight (unpublished data from the full drug checking database). The cocaine sample was highly acidic with a pH of 3 (e.g., grapefruit juice).

## Discussion

Our respondents reported that tranq dope burns when injected and used the term ‘tranq burn’ to describe the wounds it causes. Tranq dope-related SSTW etiology partially follows the “fire in the vein” hypothesis of Ciccarone and Harris (Ciccarone & Harris, 2015) wherein 1) vein loss occurs through the injection of caustic substances; this leads to 2) accidental, i.e., ‘missed shots,’ and purposeful, i.e., ‘skin-popping,’ injections into surrounding tissue, which in turn leads to 3) inflammation and possible infection if an infectious agent is introduced. The data presented in this report supports the first two conditions, but the third requires an alternative angle.

Causticness can be approximated by the feeling of how much each substance “burns” when injected, with our respondents estimating this hierarchy: cocaine burns more than xylazine/fentanyl, which in turn burns more than heroin (in powder form). PWUD specifically blamed tranq for rapid vein loss. This was also evidenced by reports of frequent missed shots, increased number of pokes per successful venous injection, increased time searching for veins, vein hardening, and especially the riskier use of large central veins for injection, including the jugular, brachial, and axillary veins. A minority of participants also reported muscling or skin-popping practices that were not frequently reported in the pre-fentanyl era in Philadelphia but were acknowledged as a contributor to SSTI (Harris, Richardson, Frasso, & Anderson, 2018).

The lab-based pH results corroborate the street-based notions of causticity, showing moderate to high acidity overall, with cocaine being the most acidic, followed by fentanyl and then xylazine. It is likely that the salt form of xylazine and fentanyl, whether hydrochloride or citrate, plays a role; however, further lab studies utilizing spectroscopy are needed. Future studies with greater sample sizes can more precisely estimate the contributions to differences in pH by chemical type, weight, volume, ratio of fentanyl to xylazine, and combinations including cocaine.

Cocaine is likely an important etiological agent based on reports of accelerated vein loss both before and during the tranq era and the suggestion of high acidity based on one lab sample with a pH of 3 in this study. In a study of six cases of xylazine attributed wounds in the Philadelphia area, laboratory confirmation of xylazine was seen in four cases and cocaine in five cases (Wei et al., 2023). Stimulant co-use with heroin/fentanyl, with and without xylazine, is on the rise nationally, with concomitant rises in mortality due to cocaine and methamphetamine (Ciccarone, 2021; Mattson, 2021). Participants in this study reported cocaine co-use to enable a more substantial ‘rush’ in the setting of tranq, one they found lacking from the xylazine component, while the fentanyl component was less pleasurable than in the past or compared with heroin. Co-use of cocaine also helps counter the sedation from tranq. The average pH of these speedballs is likely lower than tranq dope alone, a hypothesis that can be tested in future studies. Other reports highlight protective aspects of methamphetamine co-use with fentanyls sought after by consumers (Ondocsin, Holm, Mars, & Ciccarone, 2023; Mars et al., 2024). Stimulant use can also lead to formication and subsequent skin injury.

The third component of the SSTW hypothesis following venous sclerosis and compelled subcutaneous injection is more likely to be necrosis than infection in the case of tranq (Rose et al., 2023; Retrouvey et al., 2022). Our clinical observations and participants’ reports support this. Some secondary infections may occur given the exposed tissue, but an infectious agent is not considered the primary etiology (Wei et al., 2023). A synergistic etiology thus seems plausible: the more acidic components of the drug combination, cocaine and fentanyl, lead to venous sclerosis (and then the negative feedback loop of risker injections e.g. skin-popping), and the alpha agonism from the xylazine component causes peripheral vasoconstriction, leading to reduced tissue perfusion and devitalization of soft tissues.

Our observations and interviews highlight the impressive physical and psychological suffering caused by tranq-related wounds. Our findings and hypothesis extend findings of published clinical studies regarding the severity of wounds (Howard-Williams & Tierney, 2024), including those leading to limb amputation (Soderquist et al., 2024). In a large case series characterizing these wounds, many exhibited devitalized tissues and exposed deep structures with larger and more necrotic beds with chronicity (Lutz et al., 2025). In a study on the psychosocial aspects of tranq related wounds, most endorsed worry about limb loss (83 %) and wound shame (82 %), while 65 % reported delaying care (Kelly et al., 2025). Strikingly, half of the participants in this study believed it was easier to self-treat their skin wounds than seek medical attention (Kelly et al., 2025). PWUD report severe withdrawal symptoms from xylazine (Reed et al., 2025), and this often delays care seeking (Kelly et al., 2025). More research is needed to increase engagement and define best clinical practices to care for persons suffering from these wounds.

Smoking as a route of administration of fentanyl is rising nationally (Karandinos et al., 2024). In places such as San Francisco, where it is common, PWUD report ease of use, relief from needing to inject, and good bioavailability as reasons to smoke (Ciccarone et al., 2024). Xylazine is found at low levels among patients entering opioid use disorder treatment in San Francisco; however, the implications of smoking tranq are unclear. The few people we interviewed in Philadelphia who only smoked reported no, or only minor, wounds attributed to tranq. On the other hand, our respondents reported symptoms consistent with respiratory tract mucous membrane trauma due to snorting tranq. Further studies comparing the risks and benefits of smoking and injecting fentanyl and tranq are needed.

The acidity plus vasoconstriction hypothesis stemming from this research merits additional study. Animal models may lead to insights regarding risk and protective, e.g., smoking vs injecting, factors. We need to identify the contributions of the separate etiologic components, including whether people injecting fentanyl and cocaine in combination and without xylazine suffer from vein loss and SSTW. Real-world pH testing on drug solutions, as prepared by PWUD, is an essential next step.

This study has several limitations. Ethnography is limited by sampling, self-report, and recall biases; however, the contextual thick descriptions obtained may allow for greater hypothesis development and improved causal inferences for future epidemiological studies (Messac et al., 2013). These hypotheses and inferences can be tested, e.g., in population studies and animal experiments. The small sample size limits the laboratory pH testing. In addition, these in vitro tests may not represent realistically prepared street drug concentrations nor reflect the common combination of substances into a single dose. Real-world drug preparation metrics, e.g., the weight of the drug, the volume of water, and the amount of heating, may be simulated in the lab to create more realistic pH testing.

## Conclusion

‘Tranq burn’ describes the sensation and result from the injection of the fentanyl/xylazine combination. The findings in this paper lend early support to a synergistic hypothesis of tranq-related SSTW etiology: vein loss and subcutaneous injections stem from injection of acidic drugs followed by poor tissue perfusion and necrosis from injection of the vasoconstrictive xylazine. Further studies are needed to confirm this. Potential interventions include the addition of alkaline substances like baking soda for buffering and the dilution of injected drug solutions; smoking as an intervention needs further investigation.

## Figures and Tables

**Table 1:** Demographics.

Participant Information *(n* = 32)		
Gender		
	Male	18
	Female	14
Age (years)		
	20–29	8
	30–39	15
	40–49	8
	50–59	1
Race/Ethnicity		
	White	21
	Black/African American	5
	Hispanic/Latino	2
	Asian American or Pacific Islander	1
	Multiracial	3
Substances used (past month)*		
	‘Tranq’	31
	Cocaine	12
	Crack	11
	Methamphetamine	13
	Benzodiazepine (e.g. Xanax)	5
	PCP	2
	K2	2
‘Tranq’ mode of use*		
	Inject	23
	Smoke	8
	Snort	5
Presence of wounds		22
*Combined* ‘tranq’ and stimulant use		
	‘Tranq’ + Cocaine (Speedball)	12
	‘Tranq’ + Crack	1
	‘Tranq’ + Methamphetamine (Goofball)	4
	*Consecutive* ‘Tranq’ and stimulant use	6

* Numbers add up to greater than the number of participants due to polysubstance use or multiple modes of use.

**Table 2:** Laboratory pH analysis of street drug samples.

Sample	Type	Confirmation Results	weight (mg)	Volume (uL)	pH
“Dope” only (heroin, fentanyl, but no xylazine)					
1	Dope	Fentanyl (32.5%, 1p), 4-ANPP (11.1%, 0.3p), Phenethyl-4-ANPP (trace), Ethyl-4-ANPP (trace)	80.1	300	2.1
2	Dope	Heroin (16 %, 1p), 6-MAM, Acetylcodeine, Fentanyl (10.4%, 0.4p), 4-ANPP (2.1%), Quinine (trace)	125.9	400	3.6
“Tranq dope” (heroin, fentanyl, and xylazine)					
3	Tranq dope	Fentanyl (2.3%, 1p), Xylazine (59.9%, 40.9p), 4-ANPP (1.1%, 0.5p), Bupropion (0.2p), Quetiapine (0.2p)	23.6	150	5.9
4	Tranq dope	Fentanyl (5.1%, 1p), Xylazine (55.6%, 16.3p), 4-ANPP (0.7%, 0.1p), para-Fluorofentanyl (0.7%), N-Desethyl Isotonitazene (trace), Bromazolam (trace), Phenethyl-4-ANPP (trace)	26.1	150	4.9
5	Tranq dope	Fentanyl (12.9%, 1p), Xylazine (43.9%, 3.8p), 4-ANPP (3.6%, 0.4p), Phenethyl-4-ANPP (trace)	79	300	3.9
6	Tranq dope	Fentanyl (24.4%, 1p), Xylazine (37.7%, 1.4p), 4-ANPP (3.0%, 0.1p), Phenethyl-4-ANPP (trace), Ethyl-4-ANPP (trace)	32.6	150	3.6
Xylazine from lab sources					
7	Xylazine (China)	Xylazine (82.4 %)	23.8	150	5.6
8	Xylazine (Vet source)	Xylazine (71.1 %), Methylparaben, Propylparaben	24	150	4
Other drugs					
9	Coke	Cocaine (64.3 %)	119.8	400	3
10	Meth	Methamphetamine (78.3 %)	182.2	600	5.7

‘p’ refers to relative parts per sample.

## Footnotes

1 The pH scale runs from 1 to 14; 7 is neutral, 1 is extremely acidic, and 14 is extremely basic. This is a log scale, where a pH of 3 is 10× more acidic than a pH of 4.
